# PET/CT variants and pitfalls in malignant melanoma

**DOI:** 10.1186/s40644-021-00440-4

**Published:** 2022-01-04

**Authors:** Nicolas Aide, Amir Iravani, Kevin Prigent, Diane Kottler, Ramin Alipour, Rodney J. Hicks

**Affiliations:** 1 PET Centre, University Hospital, Service de Médecine Nucléaire, CHU de Caen, Avenue Côte de Nacre, 14000 Caen, France; 2 Mallinckrodt Institute of Radiology, Washington University School of Medicine, Saint Louis, USA; 3 Dermatology Department, University Hospital, Caen, France; 4 Peter MacCallum Cancer Institute, Melbourne, Australia; 5 The Sir Peter MacCallum Department of Oncology, University of Melbourne, Melbourne, Australia

**Keywords:** Melanoma, Pitfalls, variants, FDG, PET, Immunotherapy, PET technology, COVID19

## Abstract

^18^F-FDG PET/CT plays an increasingly pivotal role in the staging and post-treatment monitoring of high-risk melanoma patients, augmented by the introduction of therapies, including tyrosine kinase inhibitors (TKI) and immune checkpoint inhibitors (ICIs), that have novel modes of action that challenge conventional response assessment. Simultaneously, technological advances have been regularly released, including advanced reconstruction algorithms, digital PET and motion correction, which have allowed the PET community to detect ever-smaller cancer lesions, improving diagnostic performance in the context of indications previously viewed as limitations, such as detection of in-transit disease and confirmation of the nature of small pulmonary metastases apparent on CT.

This review will provide advice regarding melanoma-related PET protocols and will focus on variants encountered during the imaging of melanoma patients. Emphasis will be made on pitfalls related to non-malignant diseases and treatment-related findings that may confound accurate interpretation unless recognized. The latter include signs of immune activation and immune-related adverse events (irAEs). Technology-related pitfalls are also discussed, since while new PET technologies improve detection of small lesions, these may also induce false-positive cases and require a learning curve to be observed. In these times of the COVID 19 pandemic, cases illustrating lessons learned from COVID 19 or vaccination-related pitfalls will also be described.

## Background

Epidemiological studies report a rapid increase in the incidence of melanoma over the past 50 years or so, primarily in Caucasians, despite some slowing of the rate of increase around 1990–2000, probably reflecting increased awareness of the risk of excessive sun exposure and consequent UV damage to DNA. Unlike other solid tumors, melanoma mostly affects young and middle-aged people. The median age at the time of diagnosis of melanoma is 57 years [1]. However, in Australia, which has one of the highest incidences of melanoma in the world, the rate of new diagnoses increases with age, particularly in males. Close to 80% of cases present with early-stage disease (https://ncci.canceraustralia.gov.au/diagnosis/distribution-cancer-stage/distribution-cancer-stage) and have high survival rates. High-risk melanoma represents a major burden on society through both direct costs and loss of productivity.

### Mucosal/cutaneous vs uveal melanoma

Less than 5% of all primary melanomas arise from sites other than the skin. These include mucosal surfaces, meninges and the choroidal layer of the eye, which have a common ectodermal origin [2]. Mucosal melanomas can arise in the nasopharynx, larynx, tracheobronchial tracts, esophagus, anorectal and genitourinary tracts. Approximately 50% affect the head and neck region with a predominance in the sinuses and nasal cavities [3]. Mucosal melanomas are considered to be more aggressive than their cutaneous counterparts, possibly linked to delayed diagnosis with larger tumor masses, and metastatic extension at diagnosis (30%).

Uveal melanoma is a distinct clinico-pathological entity, differing in many aspects from cutaneous melanoma, including a distinct set of associated mutations [4, 5]. Its clinical course is unpredictable and metastatic disease can develop after a long disease-free interval.

Approximately 3% of metastatic melanomas are of unknown primary [6]. This potentially reflects spontaneous regression of the primary as a result of innate immune responses.

### Pattern of spread of melanoma

The main cause of death in melanoma patients is widespread metastasis. Metastases develop in regional lymph nodes, as satellite or in-transit lesions, or in distant organs. In-transit metastases occur in 5 to 8% of patients with high-risk melanoma of the limb and can present as single or multiple (sub) cutaneous nodules close to the primary tumour (satellitosis if < 3 cm from the primary tumour) or scattered over the whole extremity [7]. They can appear synchronously together with the primary tumour or as a regional relapse, and frequently precede the appearance of systemic metastases [8]. Prolonged survival can occur following locoregional therapies [9].

When it comes to distant metastases, autopsy cases have shown that multiple organ metastases were present in 95% of the patients, the most common organs involved being lymph nodes (73.6%), lungs (71.3%), liver (58.3%), brain (49.1%), bone (48.6%), heart (47.2%), adrenal glands (46.8%), and gastrointestinal tract (43.5%) [10]. A varying percentage (14% [11] to 67% [12]) of patients presenting with single organ metastasis at the time of tumor relapse has been reported in large series of patients referred to surgery, though these figures were reported at a time when modern PET sytems were not routinely used for staging and restaging of melanoma patients.

Rather than spreading to regional nodes, uveal melanoma metastasizes haematogenously, predominantly to the liver [13, 14]. Metastases to the liver develop within 15 years after the initial diagnosis and treatment in approximately 50% of patients with posterior uveal melanoma [4, 5].

### PET/CT protocol

^18^F-FDG PET/CT has been proven to have high diagnostic performance for the detection of soft-tissue, nodal and visceral metastases at initial staging or during follow-up [15] and is able to identify tumor response early in the course of TKI treatment [16]. In the framework of immunotherapy, ^18^F-FDG PET/CT has the unparalleled capability of assessing tumor response on a whole-body basis and detecting signs of immune activation as well as immune-related adverse effects (irAEs) [17–22].

In addition to the usual compliance with PET tumour imaging guidelines and harmonizing standards [23], several points regarding the PET acquisition protocol need to be raised. First, while including the brain in the field of view is not routine at many PET centres, the skull base should be included, at least for therapy assessment examinations, so that immune-related hypophysitis can be detected. While MRI is the preferred method for assessing brain and leptomeningeal metastases from melanoma, the high metabolic activity of melanoma, particularly when accompanied by surrounding vasogenic oedema can make intracranial involvement readily apparent on PET and given the small incremental imaging time to acquire from the vertex of the skull, this is recommended by the authors. When the arms are elevated to improve imaging of the thorax and axillae, more of the upper limbs are also included when whole-brain imaging is acquired.

Second, accurate staging or restaging of patients with melanoma of the extremities requires a whole-body acquisition in the case of primary tumour located on the lower limbs and the arms along the body for primary tumours located on the upper limbs. Indeed, digital PET using small-voxel reconstructions [24] or modern reconstruction algorithms [25] brings an additional value for the detection of in-transit metastases in melanoma patients, by reducing the number of indeterminate findings and minimizing falsely negative scans compared to earlier PET systems. It is noteworthy that for overweight patients, hands should be positioned on the pelvis to avoid any truncation artefact of the arms at the edges of the field of view [26].

If myocardial metastases are suspected, a special diet, long fasting period and heparin can be used to suppress physiological myocardial ^18^F-FDG uptake [27].

PET reports for therapy monitoring of patients receiving immune checkpoint inhibitors (ICIs) should include therapy response according to validated criteria, presence of signs of immune activation and signs of toxicity, especially immune-related adverse events (irAEs) requiring withdrawal of ICIs and/or corticosteroids, e.g., colitis and pneumonitis. Proposals for structured PET report can be found elsewhere [17, 28, 29].

## Pitfalls related to non-malignant diseases

### Acne and furunculosis

**Fig. 1:**
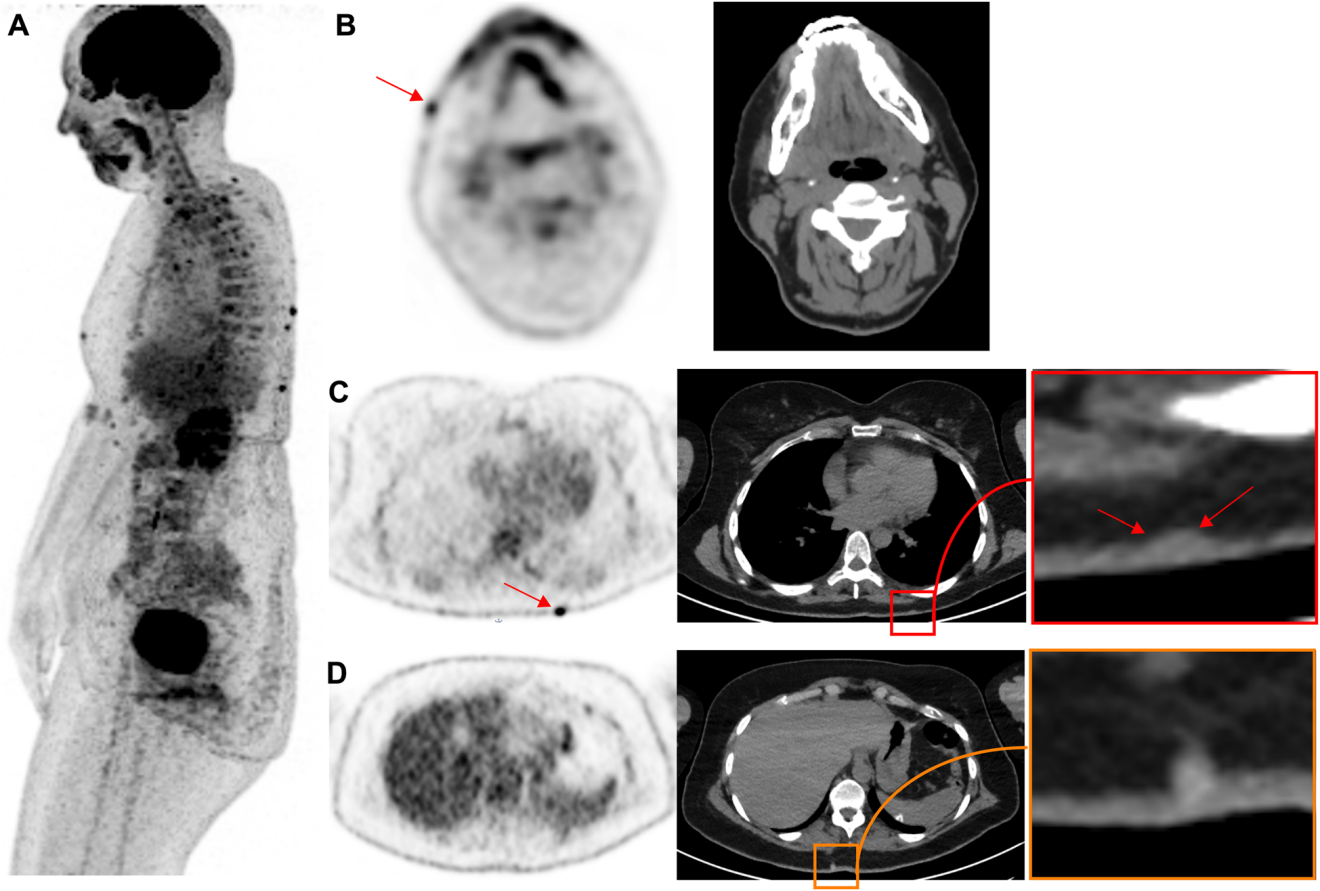
44-year-old woman, referred for initial staging for prior melanoma on the left cheek. Panel A displays lateral MIP view. Multiple subcutaneous ^18^F-FDG foci can be seen on the face (**B**) and the trunk (**C**), all located close to the midline related to dermoepidermitis. These could be confused with subcutaneous metastases without clinical correlation. It is noteworthy that some non ^18^F-FDG avid cystic lesions are also seen (**D**). Sebaceous cysts and other benign lesions apparent on correlative CT are usually readily diagnosed by clinical examination

Benign uptake related to acne and cutaneous infectious processes should not be mistaken for subcutaneous or in-transit metastases. While acne lesions tend to occur on the trunk and face, as shown in Fig. 1. Quick clinical examination before discharging the patient from the PET unit will avoid false-positive reports in the case of evident infectious skin disorders, while more complex situations may require skin examination by a dermatologist.

### Venous varicosities

**Fig. 2:**
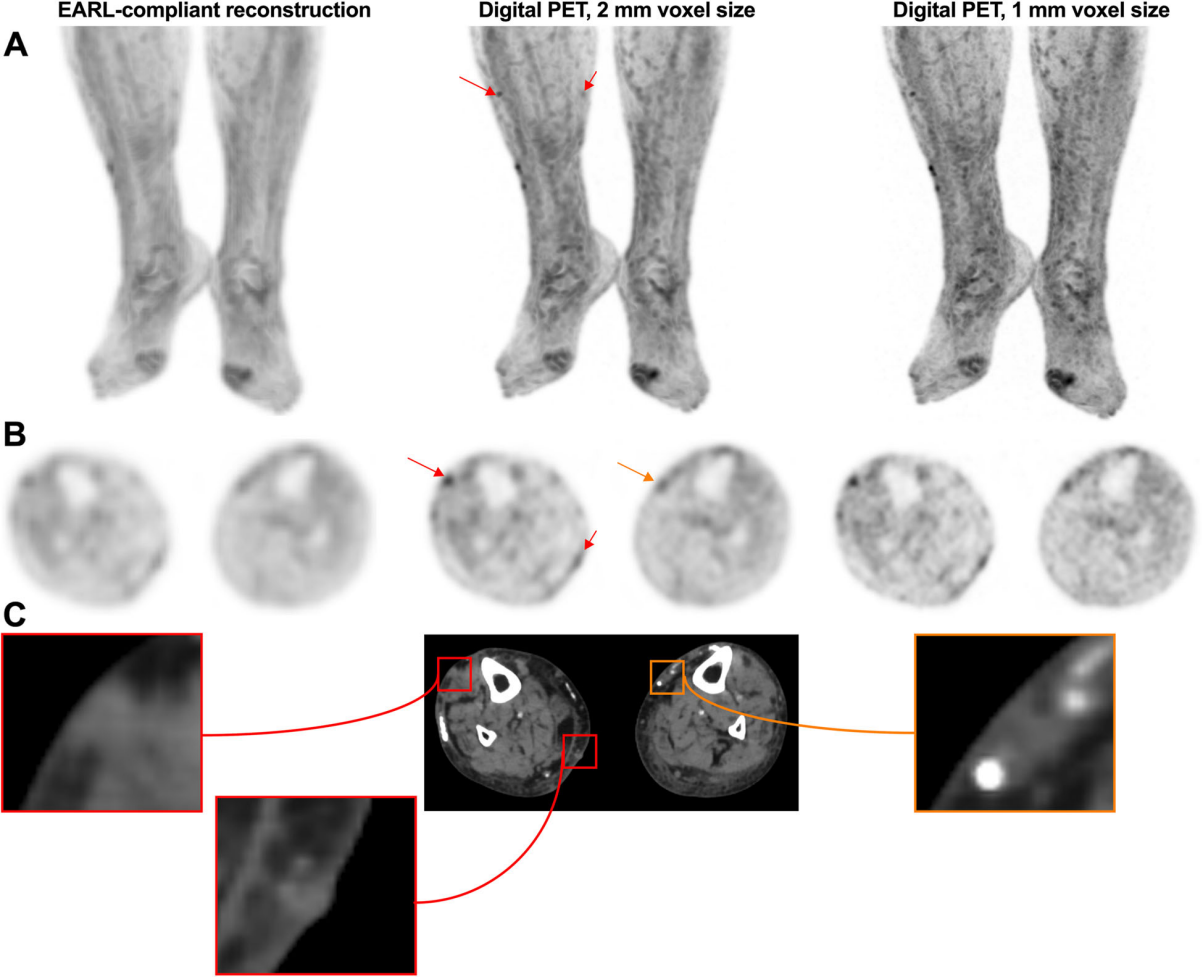
89-year-old woman with previous localization of melanoma on the right lower limb, who was on active surveillance. **A**: zoomed MIP images; **B**: transverse PET slices and **C** corresponding CT slices. Subcutaneous ^18^F-FDG foci related to in-transit metastases can be seen on the right leg (red arrows). Also visible are foci related to venous varicosity, not to be mistaken for subcutaneous metastases (orange arrow). It is noteworthy that in-transit metastases are only clearly visible on digital images reconstructed with small-voxels. EARL-compliant images, which mimic former generation PET systems by applying a post-reconstruction filtering step [23], are equivocal. EARL: European Association of Nuclear Medicine Research limited

**Fig. 3:**
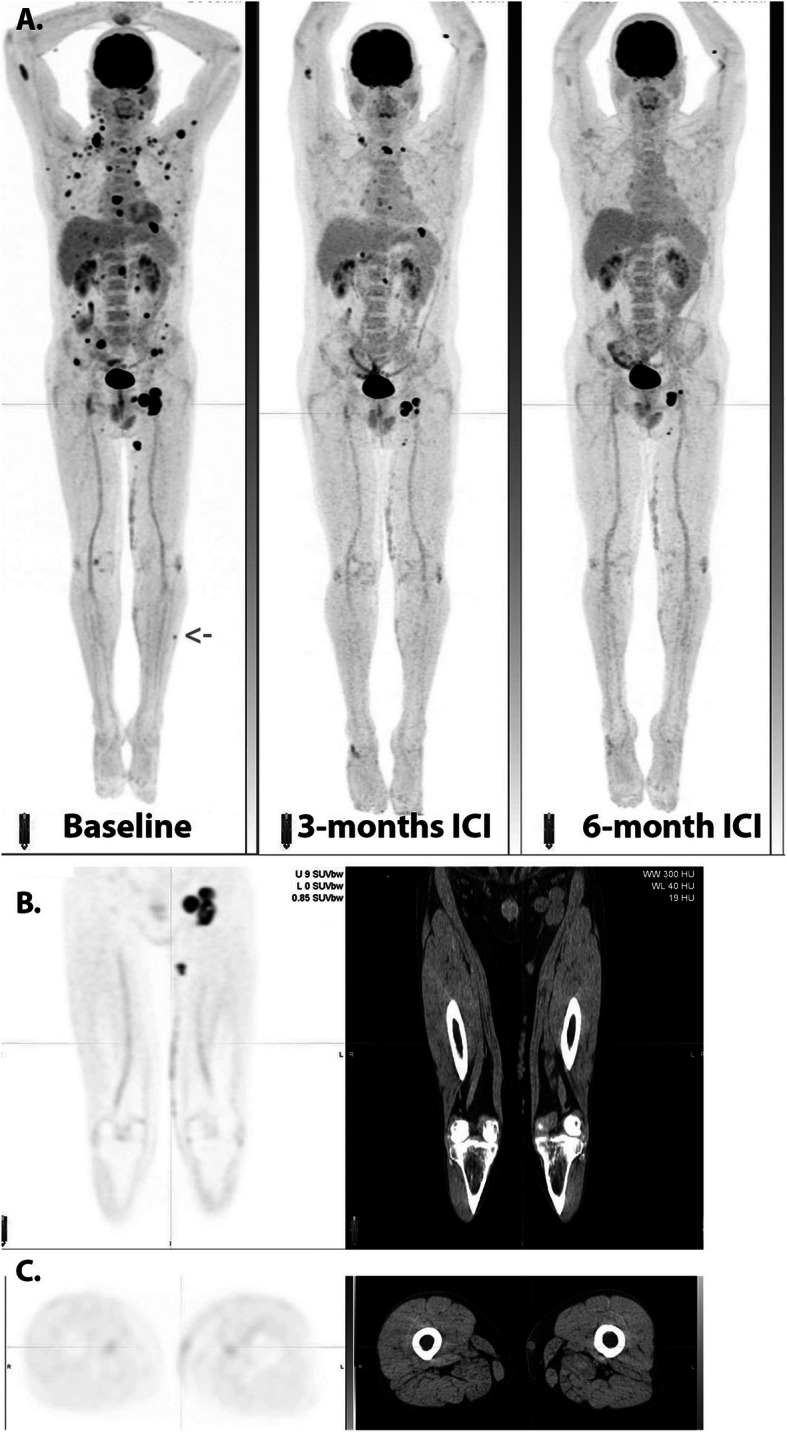
Serial MIP images (**A**) in a patient with widespread metastatic melanoma demonstrate resolution of an in-transit deposit on the lateral aspect of the left leg (arrow on baseline scan) and gradual regression of metastatic disease over time despite residual left inguinal nodal abnormality after 6 months of immune checkpoint therapy. On coronal PET and CT images (**B**), nodular but contiguous linear uptake in the subcutaneous fat along the medial left thigh demonstrates no change over time on the MIP images above and clinically correlated with a varicose vein. Transaxial images (**C**) are more difficult to interpret than the coronal images in demonstrating mildly ^18^F-FDG -avid nodularity and a rounded shape on correlative CT, emphasizing the importance of review of images in orthogonal planes

The PET reader should be aware of potential technology-related pitfalls, as new PET technologies, such as advanced reconstruction algorithms and digital PET using small-voxel reconstruction, have improved detection of small lesions [24], but may also induce false-positive cases by significantly increasing apparent uptake in benign lesions (Figs. 2 and 3).

### Post vaccine nodal uptake

PET centers scanning cancer patients after the introduction of the COVID 19 vaccination program have witnessed moderate to intense local or systemic inflammatory responses in some patients [30]. It is noteworthy that such local nodal inflammatory responses have already been observed in patients having received Influenza vaccines [31], but the frequency of these observations with COVID19 vaccine and the presence of multiple nodal uptake at higher echelon nodal stations is unprecedented.

**Fig. 4:**
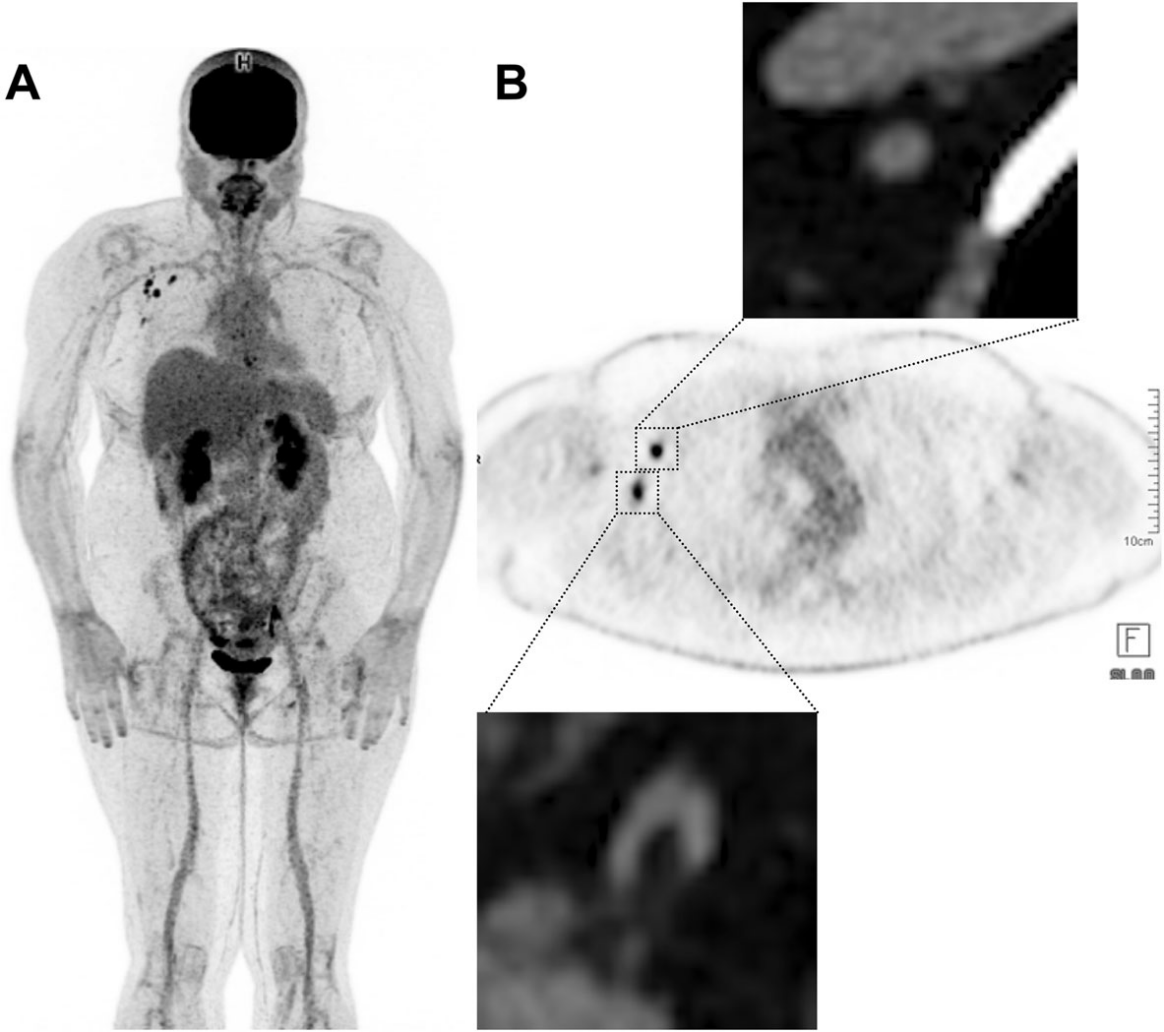
48-year-old woman with previous localization of melanoma on the left knee, who had been treated by TKI for 6 months. This patient received her first dose of COVID 19 vaccine in the right deltoid muscle (Pfizer-BioNtec Cominarty) 21 days prior to the PET examination. Multiple reactive nodes can be seen on the right axillae. Note that some of these nodes have benign CT patterns (bottom right panel: fatty hilum) while others may be considered suspicious based on CT appearance (top right panel: round shape, loss of the fatty hilum pattern). In this case, spread to the contralateral axilla from a leg primary without regional nodal or distant metastatic involvement should alert the reader to a low likelihood of malignancy

While some of them may be pretty obvious, appearing as subcutaneous and/or muscular uptake at the site of injection and contiguous nodal uptake, some other situations may be misleading for tumour uptake, especially in patients staged or followed-up for melanoma of the upper limbs. Concerning nodal uptake, it is noteworthy that foci may vary in intensity and can co-exist with both benign CT patterns (oblong shape, fatty hilum) and suspicious round shape may occur, as shown in Fig. 4. Also misleading can be the presence of benign nodal uptake in secondary echelon nodes. These lymph nodes may remain ^18^F-FDG -avid up to 10 weeks or later after vaccination [32]. For these reasons, it is prudent to adapt PET scheduling not only within treatment constraints but also accommodate the vaccination scheme of melanoma patients, as suggested recently in the largest series of vaccination-related inflammatory changes reported in the Israeli population [33].

### Sarcoid-like granuloma, especially mediastinal but sometimes systemic sarcoid

**Fig. 5:**
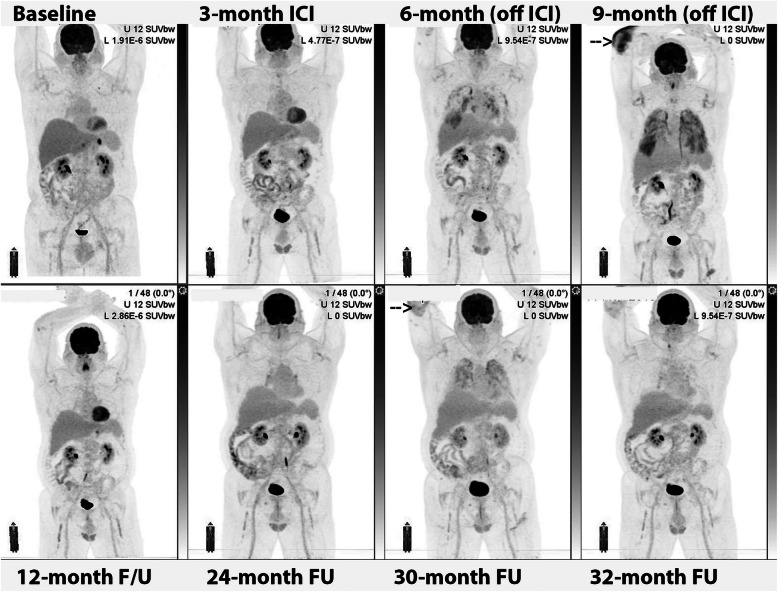
Serial MIP images. In this patient with a gastric primary and liver metastasis at baseline evaluation, the development of immune-related hepatitis led to the withdrawal of ICI treatment. Nevertheless, the patient developed pulmonary infiltrates and cutaneous lesions, particularly over the right elbow (arrow). These were confirmed on biopsy to be sarcoidosis and continued to wax and wane in response to treatment with steroids despite lack of continuing ICI treatment and ongoing complete metabolic response at prior sites of disease

Both at diagnosis and during therapy of melanoma, granulomatous disease involving nodes and other organs may cause diagnostic difficulties. Although the typical distribution of sarcoid-like lymphadenopathy includes symmetrical, generally non-enlarged hilar and mediastinal nodes with increased uptake, atypical patterns can occur. Peri-portal nodes and focal splenic lesions are not uncommon. The evolution of the sarcoid-like granulomatous disease in response to immune checkpoint therapy is highly variable and can persist beyond the withdrawal of ICIs and fluctuate in activity over time (Fig. 5).

## Technology-related pitfalls: characterization of pulmonary nodules

**Fig. 6:**
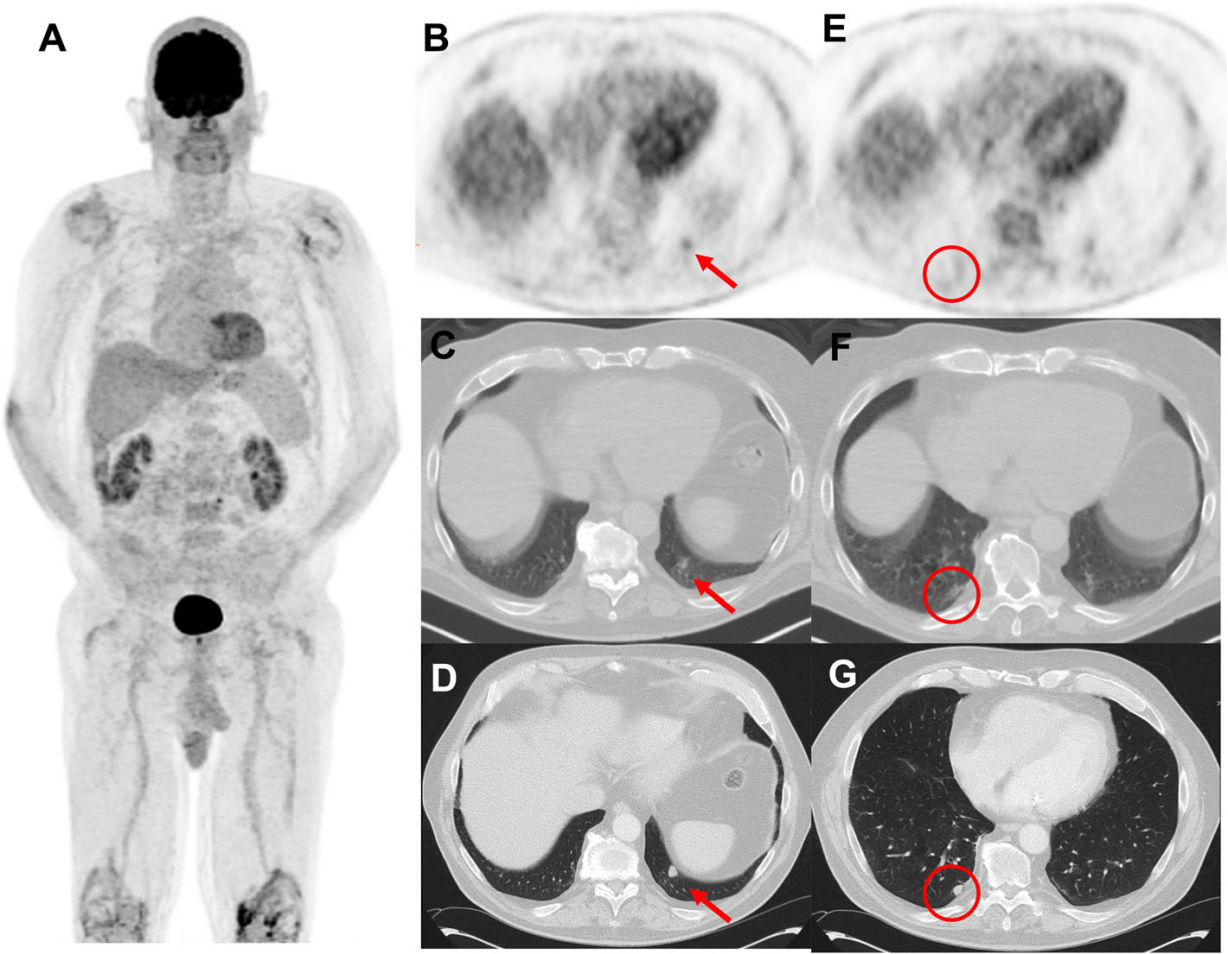
69-year-old man with prior melanoma of the right forearm, 2.1 mm Breslow-depth without ulceration and 1/mm2 mitoses and positive axillary sentinel lymph node (0.2 mm focus with no extracapsular extension) with no adjuvant therapy on observation. Panel A displays surveillance ^18^F-FDG PET MIP 5 months post-resection of the primary lesion. A new FDG-avid nodule was noted in the left lower lobe (panel B and C) which due to respiratory movement appeared ill-defined. A dedicated CT of the chest 7 days later confirmed the nodule in the left lower lobe (panel D) and also showed an additional nodule in the right lower lobe (panel G) which went undetected on the PET/CT (panel E and F) and were suspicious for metastatic melanoma. Wedge resection of the left lower lobe revealed caseation granuloma and the patient continued on surveillance

Due to respiratory movement, the ^18^F-FDG uptake and CT characteristics of the pulmonary nodules in the base of lungs may be subject to partial volume effect (Fig. 6). In these cases, a dedicated fine slice CT of the chest in full inspiration may further characterize the suspected findings. Upon detection of pulmonary nodules, other differential diagnosis needs to be considered including benign and neoplastic process and ultimately a histopathologic confirmation may be needed.

It is expected in the near future that advancements in PET motion correction will allow to better characterize small nodules located in the base of the lungs. Specifically, data-driven motion correction and the emergence of artificial intelligence tools are opening new opportunities for motion handling in clinical PET [34].

## Variants: false-negative or very low uptake in liver metastases from choroidal melanoma

**Fig. 7:**
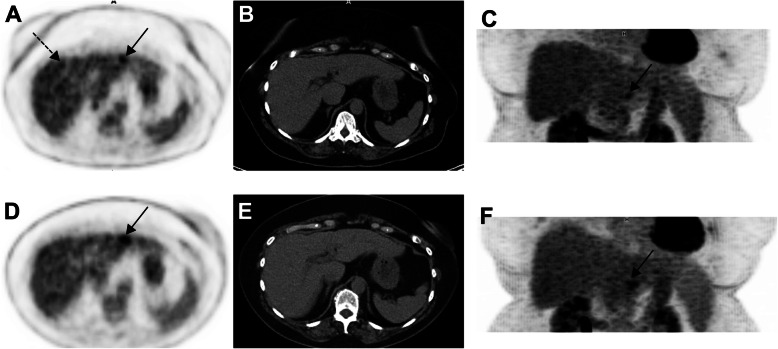
63-year-old woman with choroidal melanoma. The patient’s body mass index was 32.5. ^18^F-FDG PET images (axial slice **A**, zoomed MIP view **B**) show an indeterminate ^18^F-FDG focus located in the left liver lobe, segment 2 (black arrows) slightly more intense than the surrounding background seen in the right liver lobe (A, dotted black arrow). Given that liver metastasis is the main pattern of spread of uveal melanoma, follow-up was performed at 3 months and confirmed metastatic liver disease (**D**, **E** and **F**, black arrow)

**Fig. 8:**
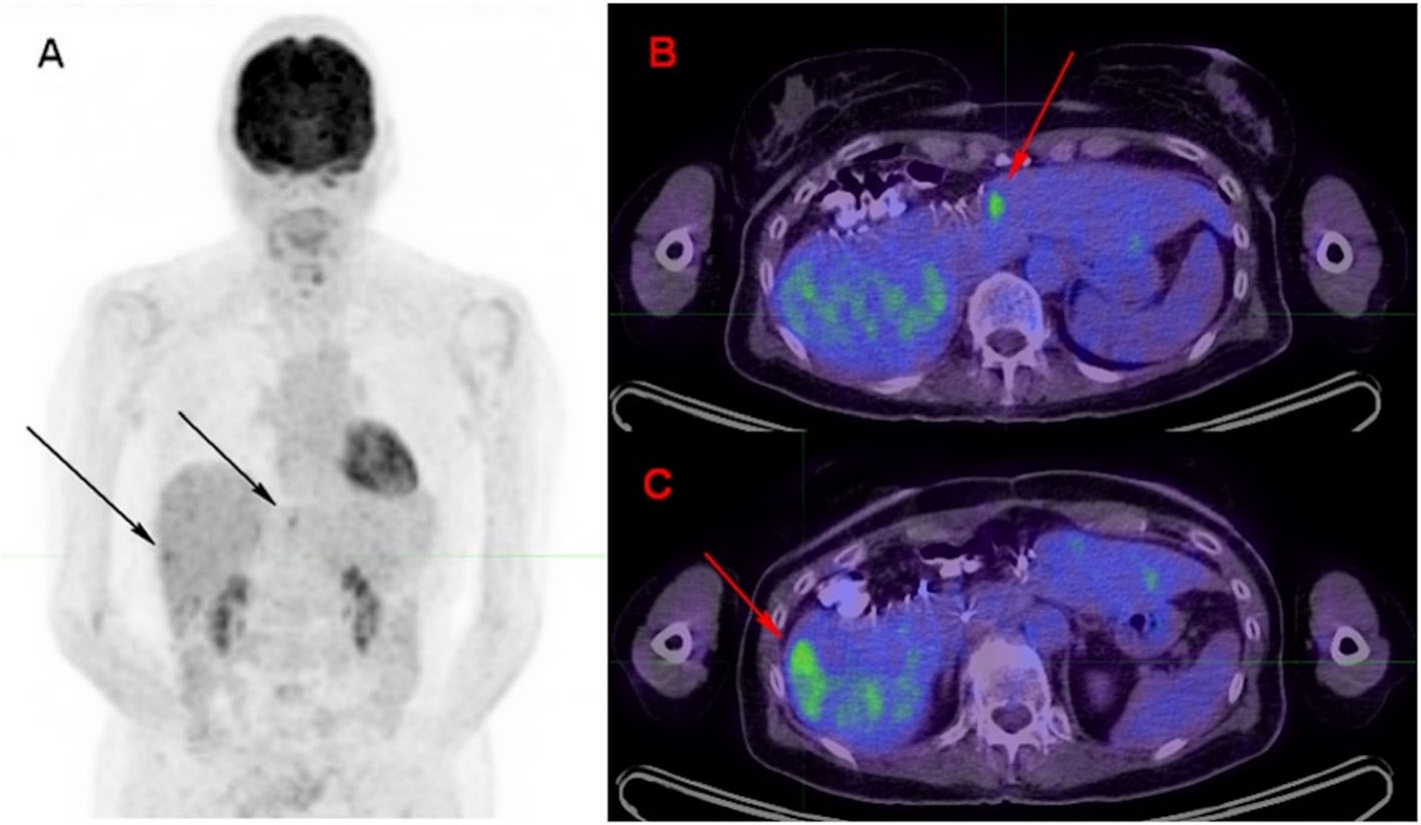
71-year-old woman with metastatic uveal melanoma, and previous central liver resection for solitary metastasis. Follow-up ^18^F-FDG PET images 6 months post-surgery showed at least two foci of low-grade ^18^F-FDG uptake in the liver, slightly above the physiological background activity (black arrows, image **A** – zoomed MIP). The lesion in segment 4a just left to the surgical margin (red arrow on image **B** - axial fused image) was subsequently confirmed to be metastatic melanoma on histology. The lesion in segment 6 (red arrow on image **C** – axial fused image), which was associated with a subtle area of hypo-attenuation on low dose CT, showed further progression on follow-up CT after 3 months (image not provided)

Uveal melanoma metastasizes haematogenously, predominantly to the liver and metastases may be of small volume. Attention should be paid to doubtful ^18^F-FDG foci, as small volume liver metastases may occur, and uptake intensity may be lower than that observed in other histological subtypes of melanoma. This is more problematic in overweight or obese patients in whom statistical considerations lead to greater inhomogeneity of apparent ^18^F-FDG uptake (as shown in Figs. 7 and 8 below), indeterminate ^18^F-FDG foci should lead to follow-up PET or additional radiological exploration. It is expected that technological evolutions such as digital PET with small-voxel reconstruction and/or enhancement of images with the convolutional neural network will further improve image quality in the liver and detection of small volume metastatic disease [34]. Because of the para-magnetic properties of melanin, MRI is a relatively sensitive technique for the detection of uveal melanoma metastases to liver and should be recommended in the setting of clinical suspicion or equivocal PET/CT findings.

## Pitfalls related to treatment

### Frequently encountered patterns

#### Colitis

Colitis may be a life-threatening irAE, requiring withdrawal of ICI and/or treatment with corticosteroids. It is noteworthy that colitis often presents as intense circumferential uptake without wall thickening and/or fat stranding on CT, and should therefore not be confused with benign uptake due to metformin treatment in diabetic patients.

**Fig. 9:**
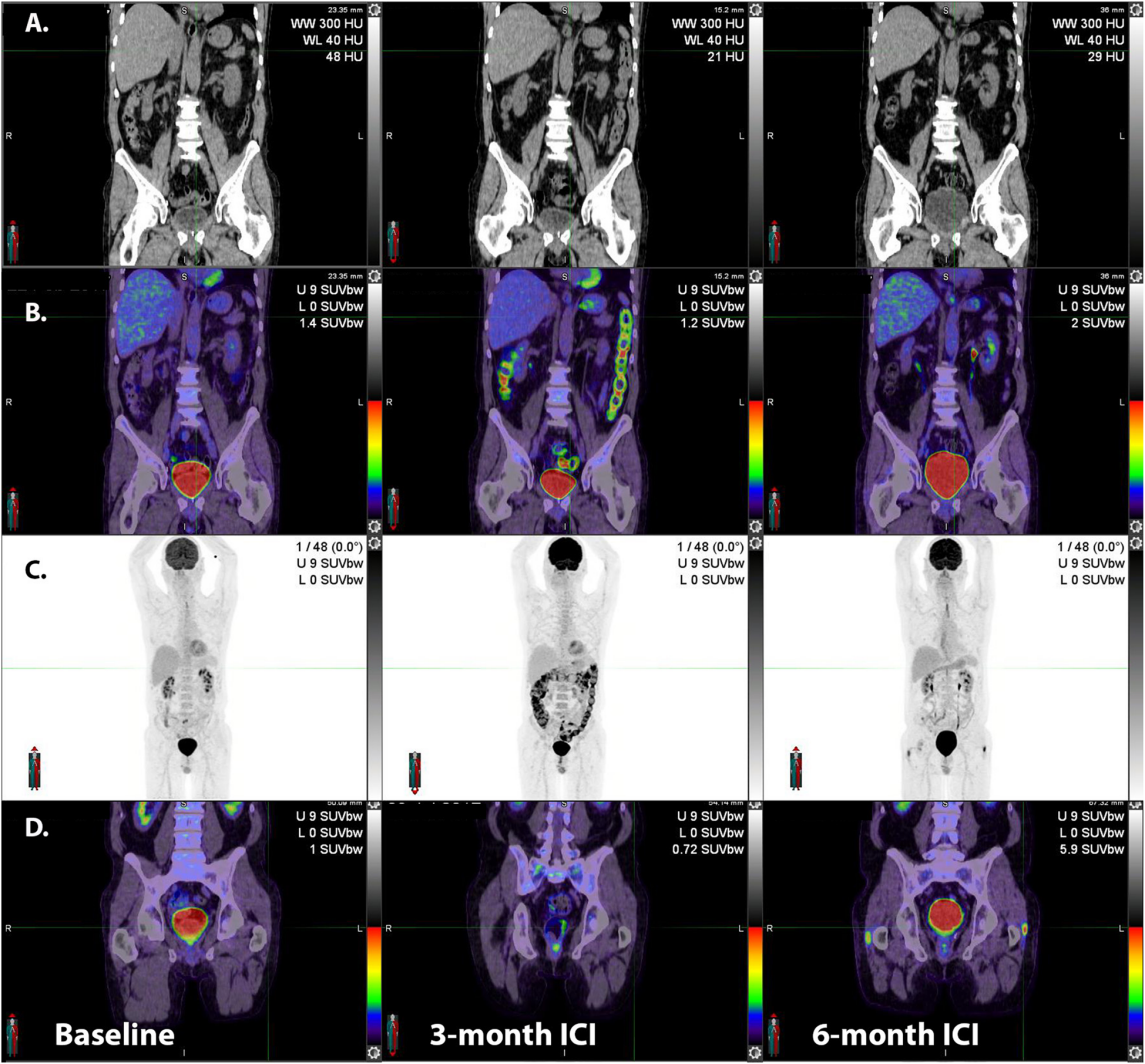
Following the introduction of adjuvant immunotherapy for resected stage IIIC disease, despite normal CT appearances (**A**, middle panel), fused ^18^F-FDG PET/CT (**B**, middle panel) and MIP (**C**, middle panel) images revealed intense uptake throughout the large bowel with a typical “string of pearls” distribution. The patient was asymptomatic at the time of the scan but within 24-h had developed severe diarrhea consistent with this diagnosis. Despite successful treatment of this complication the patient subsequently developed bilateral trochanteric bursitis as a second potential irAE

A typical feature of immune-related colitis is prominent of the haustra as well giving rise to an appearance similar to a “string of pearls”. Unlike physiological bowel activity, which is often segmental, the entire colon is generally involved by autoimmune colitis. It should be noted that immune-related adverse events can occur simultaneously or be temporally unrelated (Fig. 9).

#### Diffuse enteritis masking small bowel and serosal metastases

**Fig. 10:**
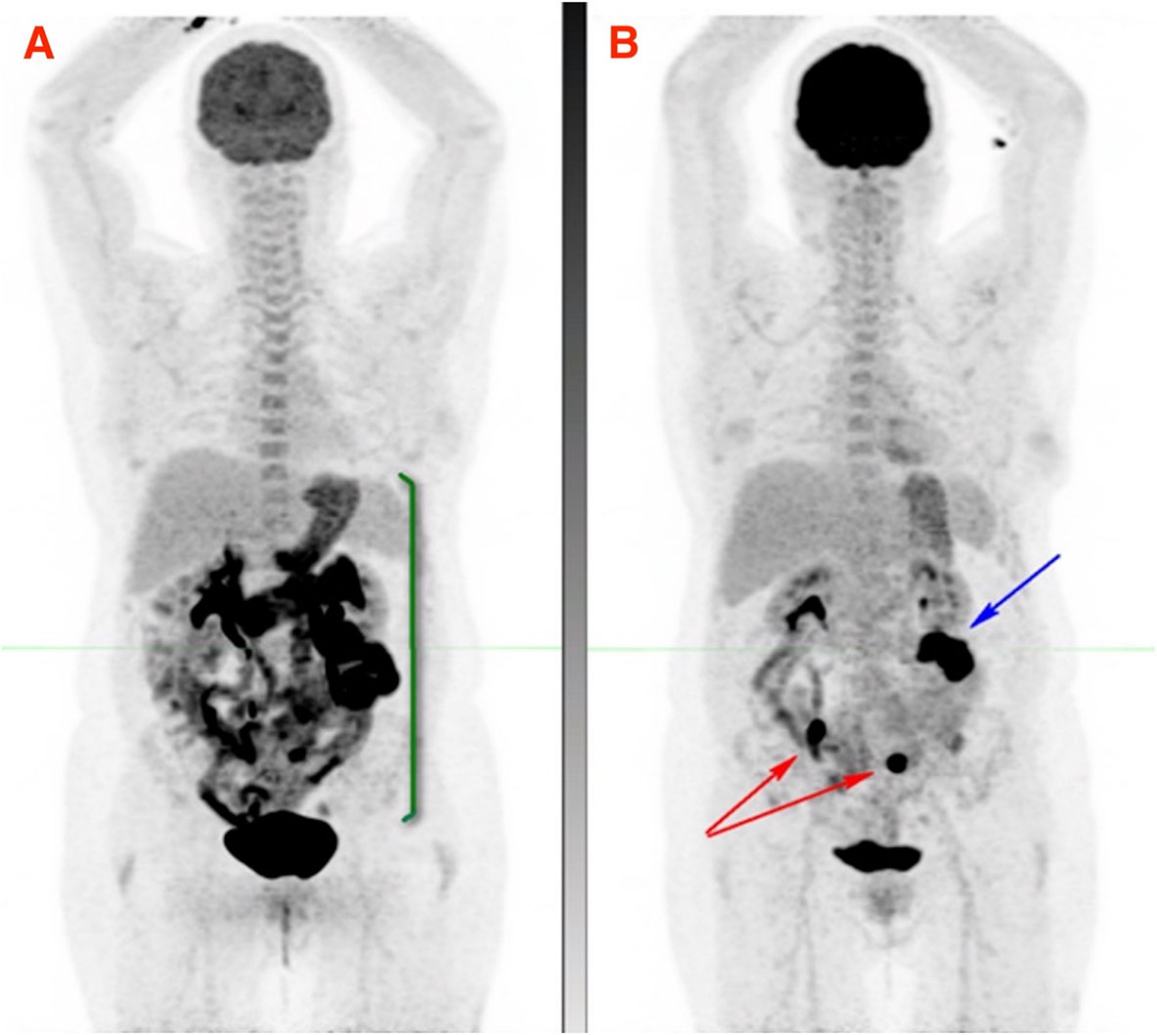
Patient with stage 4 melanoma in CMR on single-agent nivolumab with symptoms of intermittent nausea, vomiting and diarrhoea and clinical diagnosis of immune-related enteritis underwent ^18^F-FDG PET which showed moderate to intense diffuse uptake in the stomach, duodenum and small bowel in keeping with immune-related gastritis, duodenitis and enteritis (MIP image **A**). Short interval ^18^F-FDG PET after successful treatment of irAEs (MIP image **B**), showed two foci of intense FDG uptake in the small bowel (red arrows) in keeping with metastases and a third focus in the left abdomen near the transverse colon, in keeping with serosal metastasis (blue arrow)

Small bowel is a common site for gastrointestinal metastasis in melanoma, however, the history and clinical findings are nonspecific and may mimic immune-related gastritis and enteritis. The presence of the latter, could potentially mask small bowel metastases on ^18^F-FDG PET/CT (Fig. 10).

#### Pneumonitis

**Fig. 11:**
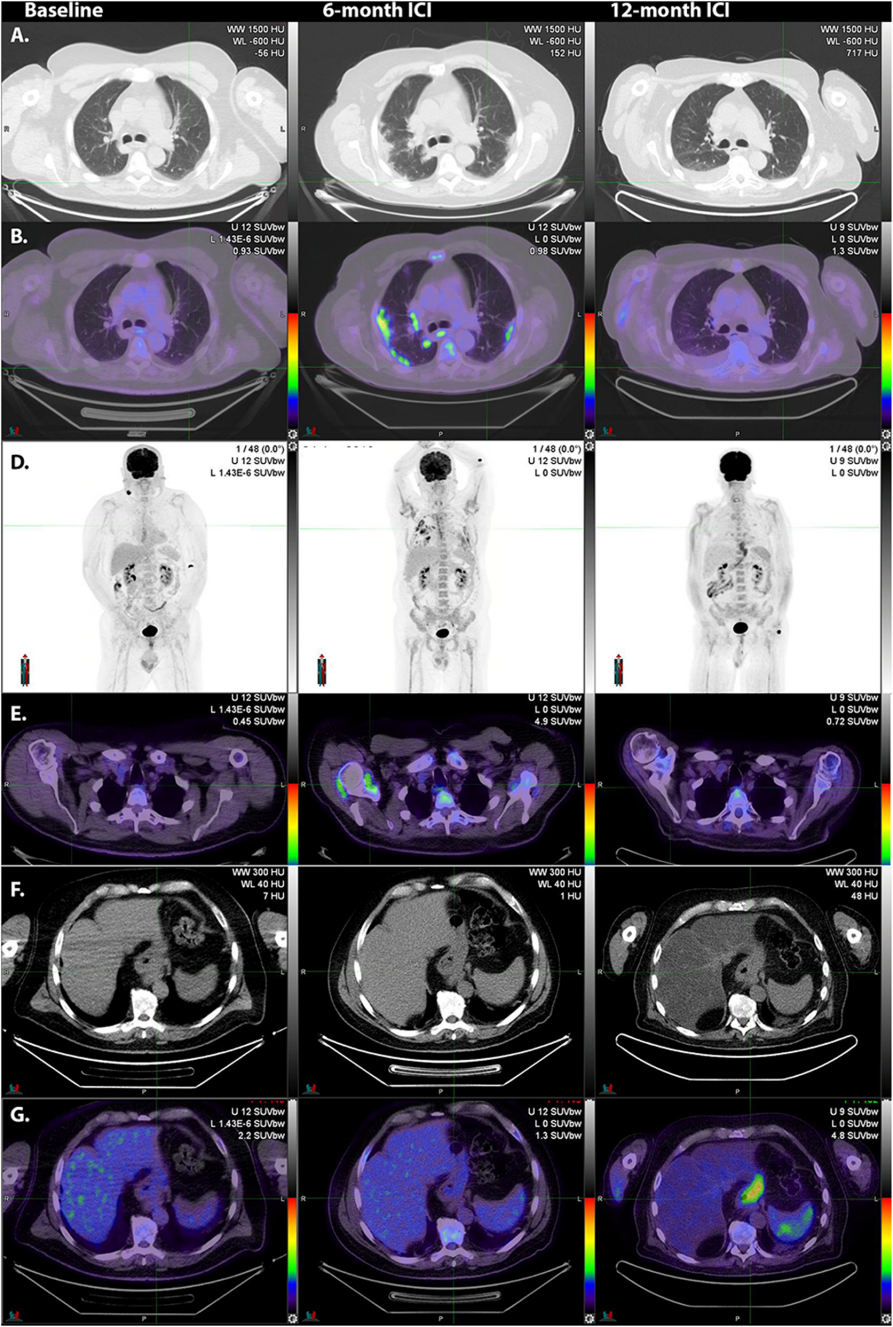
Following resection of nodal metastasis in the right lower cervical region, adjuvant immune checkpoint inhibitor therapy was commenced. At 6-month surveillance ^18^F-FDG PET/CT, lung infiltrates were apparent on correlative CT (**A**). These had high ^18^F-FDG -avidity (**B**, middle panel). Serial MIP images (**C**) demonstrate the patchy, bilateral nature of these changes as well as generalized bone marrow activation and increased activity in both shoulders consistent with synovitis as confirmed on transaxial fused ^18^F-FDG PET/CT images (**D**, middle panel). With the introduction of corticosteroids, the pneumonitis resolved but a fatty change was noted in the liver (**F**, right panel) and diffuse oesophagogastric uptake was observed (**G**, right panel). Gastroscopy confirmed enteritis. Increased spleen to liver ratio on the late scan likely reflects a combination of reduced hepatic activity related to steatosis and splenic activation

Along with colitis, pneumonitis (Fig. 11) may be a life-threatening irAE, requiring withdrawal of ICI and/or treatment with corticosteroids.

Although immune-related pneumonitis is the most common cause of patchy increased ^18^F-FDG uptake in the lung, other pathologies should always be considered. In the COVID-19 era, these include pulmonary manifestations of viral infection [35]. Pulmonary sarcoidosis is also a differential diagnosis but is more often nodular than of a ground-glass or diffuse subpleural distribution. Of course, both conditions may co-exist.

#### Hepatitis

**Fig. 12:**
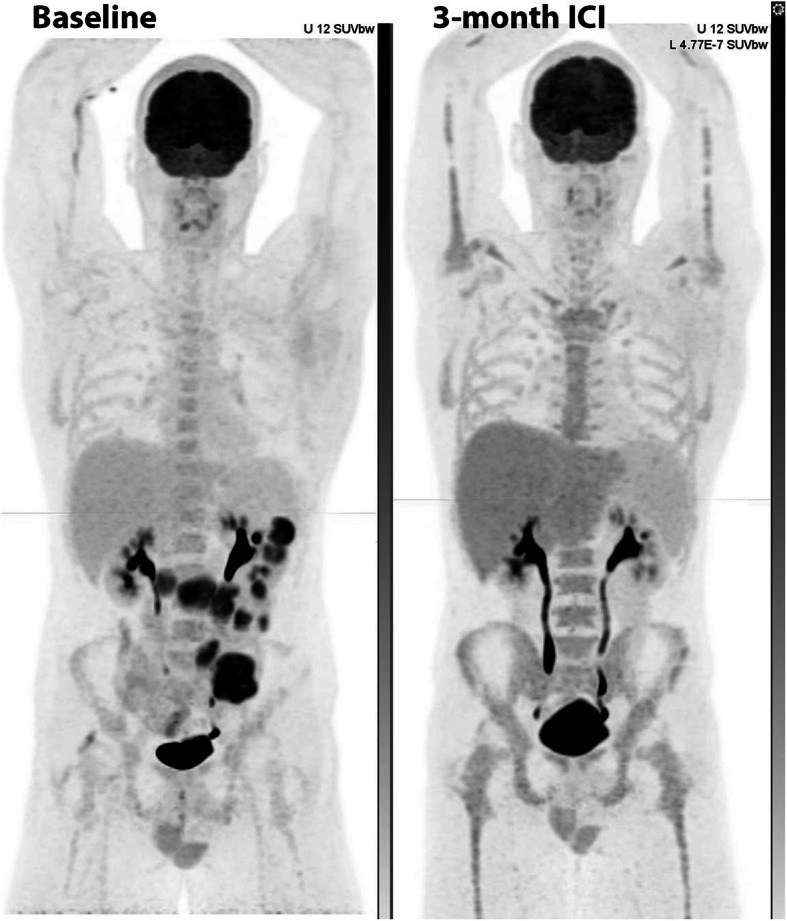
Note the relatively high uptake in the liver relative to the spleen on the post-treatment scan, which was associated with biochemical evidence of hepatitis. Note also that the generalized bone marrow activation with peripheral marrow expansion in the context of a complete metabolic response in prior small bowel metastases was interpreted as being reactive

Autoimmune hepatitis is relatively common as a side effect of ICI therapy but is usually detected on routine biochemistry surveillance. Occasionally it can persist and be apparent on ^18^F-FDG PET/CT (Fig. 12).

### Unusual irAEs during immunotherapy

#### Pancreatitis mimicking malignancy

**Fig. 13:**
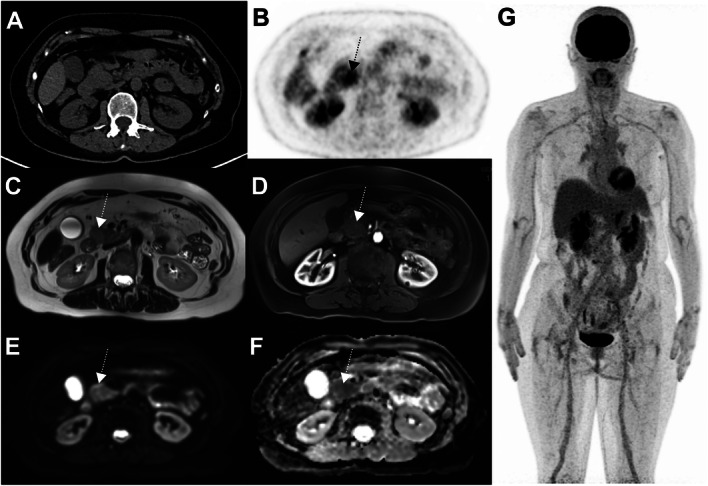
63-year-old woman with prior localization of melanoma on the left thigh, who had been treated by ICIs for 16 months. A nodular 18F-FDG focus can be seen in the pancreatic head (**B**: axial PET slice) without concordant anomaly at CT (**A**). Given that this pattern was unusual, MRI was performed a week later and showed patterns consistent with pancreatic adenocarcinoma: ill-defined mass of the pancreatic head on T2 sequence (**C**), this lesion appearing hypointense on T1 sequence arterial phase after gadolinium injection (**D**), hyperintense on diffusion sequence and displaying hypercellularity on ADC sequence. Given that PET was otherwise normal, a biopsy was performed and confirmed pancreatitis. Subsequent PET showed spontaneous recovery (data not shown)

Pancreatitis has been reported to occur in 2.7% of patients receiving ICIs, more frequently with anti-CTLA4 (3.08% versus 0.94% for anti PD1) [36]. Also, melanoma patients treated with ICIs have increased incidence of pancreatitis as compared to other solid tumours. Although not misleading when appearing as homogeneous increased ^18^F-FDG uptake involving the whole gland, pancreatitis can also appear as focal uptake mimicking either primary tumour or metastatic disease (Fig. 13).

**Fig. 14:**
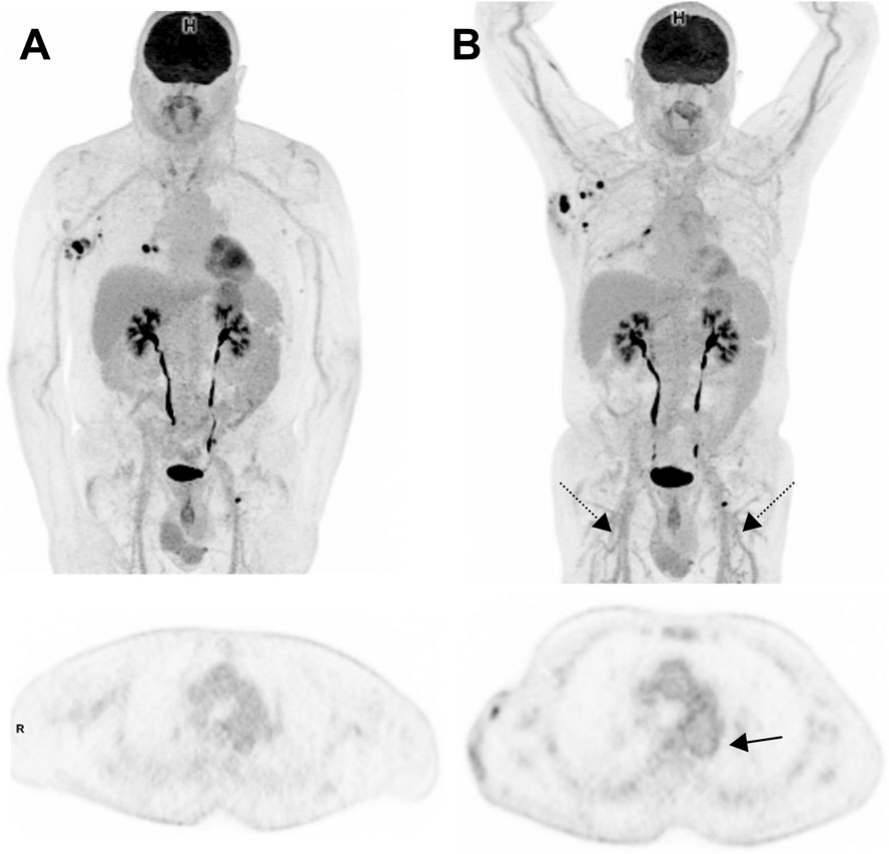
69-year-old man with prior localization of melanoma on the trunk. Baseline images (MIP view and transaxial slice at the level of the descending aorta) are shown at baseline (**A**) and interim PET (**B**). Recurrent nodal disease in the right axillae and the right hilum can be seen. Interim PET, performed at the time the patient who had been treated with ICIs for 5 months, shows the appearance of a diffuse vasculitis pattern involving not only the thoracic aorta but also brachial and femoral arteries (including small-caliber arteries, black dotted arrows) but lack of response in axillary nodal disease

Peripheral vasculitis (Fig. 14).

**Fig. 15:**
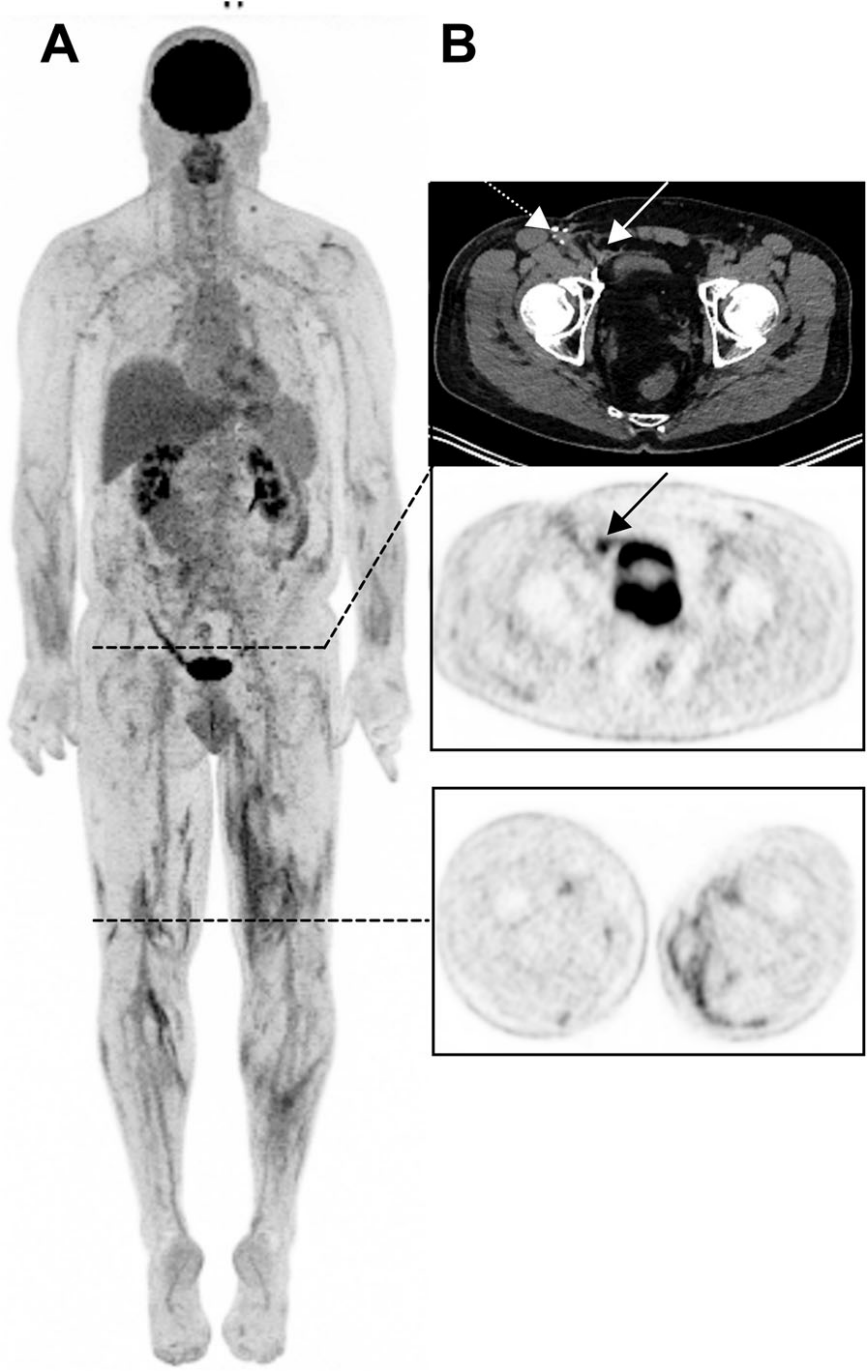
55-year-old man with an unknown primary melanoma who had been treated with ICIs for 35 months (Pembrolizumab). 3D MIP view shows multiple intermuscular foci in the lower limbs (B, lower panel). Also noteworthy is a benign uptake in the right groin (B, black arrow) related to a right inguinal mesh repair (white arrow), not to be confounded with a recurrence in the area of the lymphadenectomy (white dotted arrow)

Fasciitis (Fig. 15).

1. TKI-induced Hemophagocytic lymphohistiocytosis (HLH): Not to be confused with a “banal” inversion of the liver to spleen ratio

**Fig. 16:**
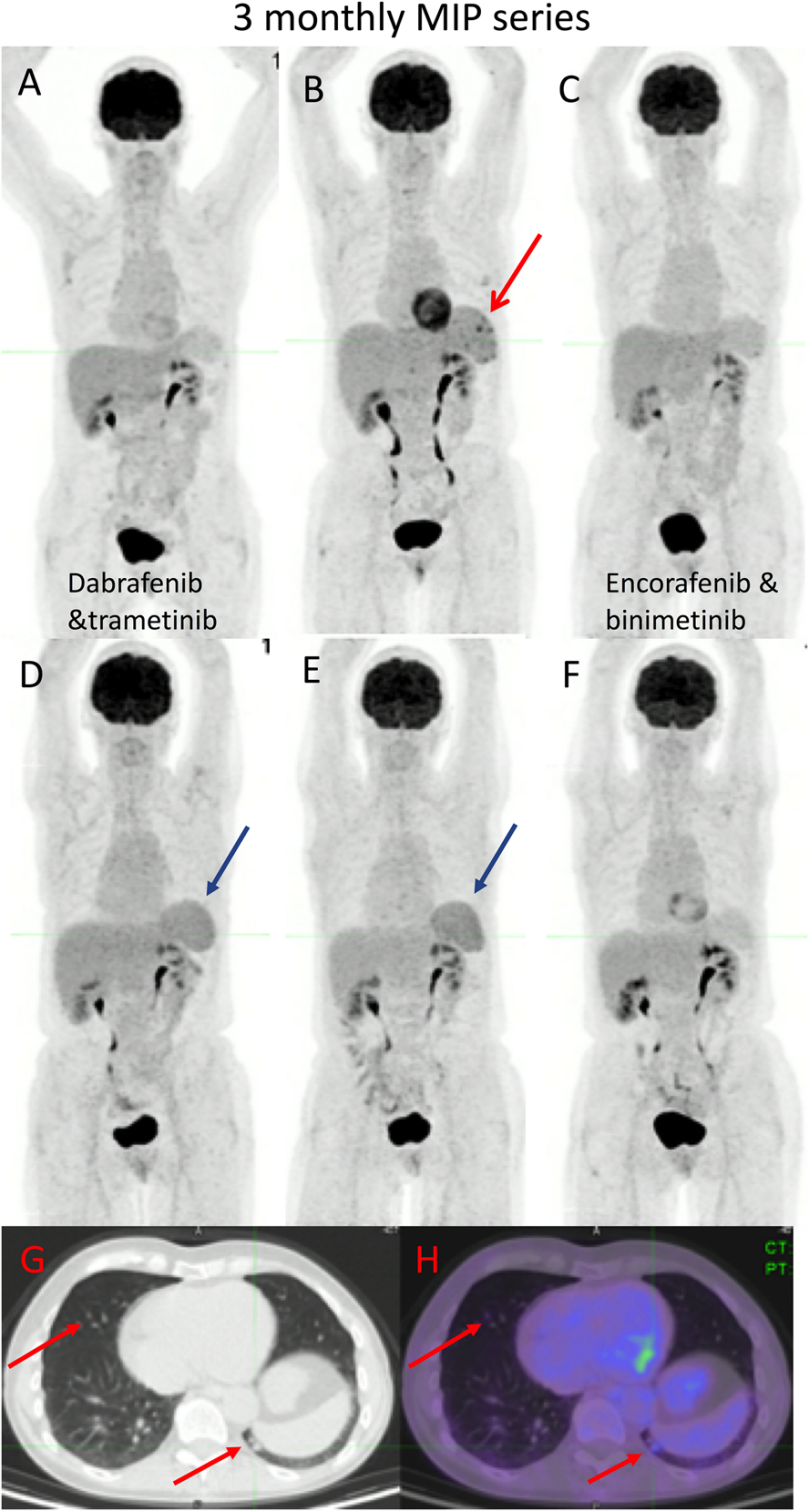
68-year-old female with primary melanoma in the upper back (1 mm Breslow thickness, no ulceration, mitotic rate < one, no peri-neural or lymphovascular invasion) with negative sentinel node biopsy at diagnosis (5 years ago) who developed self-detected right axillary metastasis one year later (images not provided). Axillary nodal dissection demonstrated 1/9 lymph nodes involved with 8 mm deposit and 0.3 mm extra-nodal extension (stage IIIB) BRAF V600K. Started on ICI (Ipilimumab and Nivolumab) afterwards and developed multifocal acquired demyelinating sensory and motor neuropathy treated with steroids, plasmapheresis and rituximab. Surveillance ^18^F-FDG PET/CT (A) showed new small pulmonary nodules (not evident on MIP),red arrows axial fused image(G and H); which were confirmed to be metastatic melanoma on wedge resection. MRI brain also showed small new cerebral metastases (stage IV-M1b). Treatment subsequently changed to Dabrafenib and Trametinib, which was poorly tolerated with recurrent episodes of fever, myalgia and fatigue. Surveillance ^18^F-FDG PET/CT scans were performed 3 monthly afterwards; the 1st follow-up ^18^F-FDG PET/CT showed an enlarged spleen with multiple foci of moderately increased FDG uptake (red arrow - B). The treatment regimen changed to 4 days on and 3 days off; however, this was also poorly tolerated and the patient was switched to Encorafenib and binimetinib with subsequent scans showing a mild interval splenic enlargement and reversal of the spleen to liver activity (blue arrows – D,E). The patient remained in CMR on subsequent scan and reversal of spleen to hepatic activity normalised

HLH is related to an excessive immune activation leading to high fever, cytopenia, hepatosplenomegaly and multi-organ injury. HLH has been reported to induce an increased splenic uptake on ^18^F-FDG PET/CT greater than that observed during sepsis [37]. HLH can be observed during treatment with BRAF/MEK inhibitors, as shown in the case below (Fig. 16), and has also been reported in patients receiving ICIs.

### Bullous pemphigoid

**Fig. 17:**
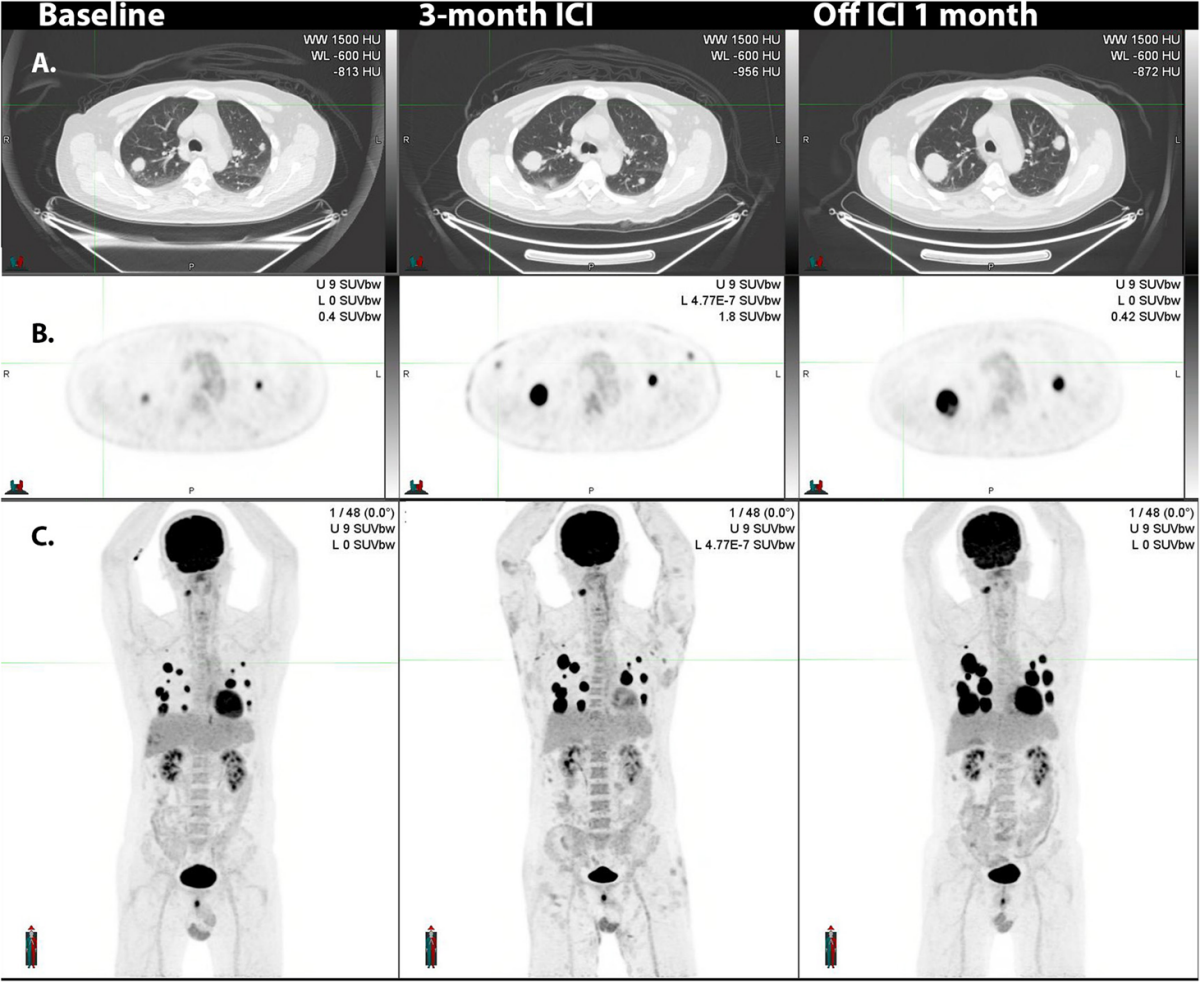
**A**: in this patient with multiple lung metastases identified at baseline evaluation, which were highly ^18^F-FDG-avid (**B**). ICI administration led to the development of widespread cutaneous lesions with associated axillary and inguinal nodal uptake apparent on ^18^F-FDG PET/CT. Biopsy confirmed bullous pemphigoid and the ICI therapy was ceased. Serial MIPs demonstrate resolution of the skin changes and nodal changes consistent with an inflammatory basis, but the progression of disease, as suggested already on both CT and PET at early evaluation

Severe autoimmune skin involvement is an uncommon but recognized complication of ICI. Bullous pemphigoid is the most common of these (Fig. 17).

### Multiorgan irAEs including nephritis

**Fig. 18:**
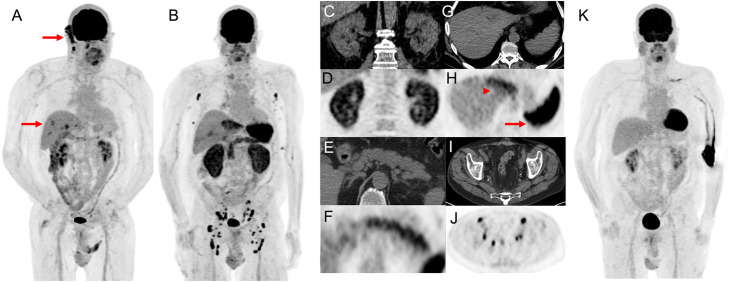
69 year-old man with ulcerated right occipital scalp melanoma, 5 mm deep, mitotic rate of 4 with locoregional nodal metastasis in the right sub-occipital and cervical stations as well as biopsy proven distant metastatic disease in the liver demonstrated on ^18^F-FDG PET MIP (arrows, panel **A**). Following three cycles of combination ipilimumab and nivolumab presented to emergency department with acute kidney injury (serum creatinine rose from 0.8 mg/dL to 2.8 mg/dL) with progressive fatigue, generalized weakness and poor oral intake. ^18^F-FDG PET MIP (panel **B**) at 3 months showed resolution of all disease in the right neck and liver and development of FDG-avidity of multiple organs including diffuse parenchymal ^18^F-FDG uptake by both kidneys (panel **C** and **D**), diffuse FDG uptake in the pancreas (panel **E** and **F**), diffuse ^18^F-FDG uptake in the left lobe of the live (arrow heads, panel **G** and **H**), marked diffuse ^18^F-FDG uptake in the spleen (arrows, panel **G** and **H**) as well as bilateral axillary, iliac and inguinal lymphadenopathy (panel **I** an **J**) suggestive of, interstitial nephritis, pancreatitis, likely cholangitis and granulomatous disease, respectively. The patient discontinued ICIs and was treated with high dose corticosteroid and all findings resolved at 5 months follow-up with gradual improvement in serum creatinine (Panel **K**)

Autoimmune renal pathologies are rare but important irAEs related to ICI treatment and normally diagnosed by routine laboratory testing but occasionally apparent on ^18^F-FDG PET/CT. Typical features are renal enlargement with increased renal parenchymal retention of tracer (Fig. 18).

## Conclusion and perspectives

Technological improvements allow us to revisit clinical problems previously viewed as limitations of PET such as detection of in-transit disease, evaluation of small pulmonary nodules apparent on CT and detection of liver metastases in choroidal melanoma, but sometimes produce artefacts and pitfalls requiring a learning curve to be observed.

Knowledge of variants and treatment-related pitfalls is crucial to avoid misinterpretation. ICIs-related pitfalls should not be regarded as a drawback of PET imaging, as they actually reflect the unique capability of PET to perform whole-body imaging and capture signs of immune activation in addition to performing therapy monitoring.

In these times of COVID 19 pandemic, PET scheduling should probably be adapted not only within treatment constraints but also accommodate the vaccination scheme of melanoma patients to avoid false-positive results. Despite this, tricky situations may occur, such as differentiating active COVID-19 related lung involvement from immune-pneumonitis. In any case, PET reporting should be made with knowledge of clinical findings (if needed quick physical examination in the PET unit may be required), laboratory and correlative morphological imaging, and obviously, collaboration with the referring physician and discussion at tumour boards remains crucial for an efficient patient’s management.

## Data Availability

Not applicable.

## Abbreviations

TKI: tyrosine kinase inhibitors
ICIs: immune checkpoint inhibitors
irAEs: immune-related adverse events
UV: ultraviolet
DNA: deoxyribonucleic acid
PET: positron emission tomography
CT: computed tomography
^18^F-FDG: Fluorodeoxyglucose
MRI: magnetic resonance imaging
MIP: maximum intensity projection
EARL: European association of nuclear medicine research limited
HLH: Hemophagocytic lymphohistiocytosis
